# Visualising tapasin- and TAPBPR-assisted editing of major histocompatibility complex class-I immunopeptidomes

**DOI:** 10.1016/j.coi.2023.102340

**Published:** 2023-08

**Authors:** Andy van Hateren, Tim Elliott

**Affiliations:** 1 Institute for Life Sciences and Centre for Cancer Immunology, Faculty of Medicine, University of Southampton, Building 85, Southampton SO17 1BJ, UK; 2 Centre for Immuno-oncology and CAMS-Oxford Institute, Nuffield Department of Medicine, University of Oxford, Old Road Campus, Headington, Oxford OX3 7BN, UK

## Abstract

Which peptides are selected for presentation by major histocompatibility complex class-I (MHC-I) molecules is a key determinant of successful immune responses. Peptide selection is co-ordinated by the tapasin and TAP Binding PRotein (TAPBPR) proteins, which ensure MHC-I molecules preferentially acquire high-affinity-binding peptides. New structural analyses have offered insight into how tapasin achieves this function within the peptide-loading complex (PLC) (comprising the Transporter associated with Antigen Presentation (TAP) peptide transporter, tapasin–ERp57, MHC-I and calreticulin), and how TAPBPR performs a peptide editing function independently of other molecules. The new structures reveal nuances in how tapasin and TAPBPR interact with MHC-I, and how calreticulin and ERp57 complement tapasin to exploit the plasticity of MHC-I molecules to achieve peptide editing.

## Highlights

• Structures of MHC-I bound by tapasin, tapasin–ERp57 or the PLC have been reported.
• Comparison with TAPBPR–MHC-I structures reveals similarities, and some differences.
• Tapasin binds MHC-I via multiple interactions distributed over a broad area.
• A loop of tapasin widens the peptide-receptive MHC-I α-helices and traps peptides.
• Peptide loading and deglucosylation of the MHC-I glycan are allosterically linked.

**Current Opinion in Immunology** 2023, **83**:102340

This review comes from a themed issue on **Antigen processing**

Edited by **Terri Laufer** and **Laurence Eisenlohr**

For complete overview of the section, please refer to the article collection, “Antigen Processing (June 2023)”

https://doi.org/10.1016/j.coi.2023.102340

0952–7915/© 2023 The Author(s). Published by Elsevier Ltd. This is an open access article under the CC BY license (http://creativecommons.org/licenses/by/4.0/).

## MHC-I peptide selection

Major histocompatibility complex class-I (MHC-I) molecules bind and present peptides to T cells and natural killer cells, protecting jawed vertebrates from intracellular pathogens and cancer 1, 2, 3•, 4. MHC-I molecules become loaded with peptides via peptide-loading complexes (PLC) in the endoplasmic reticulum. In humans, each PLC contains one or two editing modules, with each module comprising a peptide-receptive MHC-I heavy-chain–β_2_-microglobulin heterodimer linked by tapasin to the TAP transporter, with calreticulin and ERp57 completing a network of interactions [5].

MHC-I peptide loading occurs iteratively, with tapasin facilitating exchange of low- for high- affinity peptides 6, 7, 8, 9, 10, 11. Peptide-loaded MHC-I complexes then dissociate from the PLC once glucosidase II has removed the last glucose from the N-linked glycan attached to asparagine 86 (N86) of MHC-I [12], thereby removing the binding site for calreticulin. Peptide–MHC-I complexes then egress through the secretory pathway where some MHC-I molecules experience scrutiny from the tapasin homologue, TAPBPR, which like tapasin, preferentially selects optimal, high-affinity-binding peptides for presentation 13, 14, 15. Peptide-empty MHC-I molecules that are generated at this second quality control checkpoint are afforded another attempt at peptide loading following re-addition of glucose to the N-linked glycan attached to N86 of MHC-I via UDP-glucose:glycoprotein glycosyltransferase and calreticulin facilitated retrieval to the PLC 12, 16, 17, 18, 19, 20.

The genes encoding MHC-I molecules are highly polymorphic, and MHC-I allotypes differ in key attributes, including peptide specificity and dependence upon tapasin or TAPBPR for peptide acquisition 2, 15, 21, 22, 23, 24. We recently discussed how the dynamic properties of MHC-I molecules underpin peptide selection, and how tapasin and TAPBPR achieve peptide editing by modulating MHC-I dynamics [25]. Here, we discuss new structural insights into MHC-I peptide loading, including structures of MHC-I bound by tapasin or tapasin–ERp57 heterodimers, as well as high-resolution structures of an intact PLC.

## Comparisons of tapasin-bound major histocompatibility complex class-I structures

In 2017, two groups provided crystal structures of TAPBPR-bound MHC-I molecules 26, 27, while a structure of an intact detergent-solubilised human PLC was reported, albeit with lower resolution, with an editing module resolved by cryo-electron microscopy (cryo-EM) at 5.8 angstroms [5]. Despite these structures, a significant degree of uncertainty remained about how peptide editing occurred, which recent high-resolution crystal structures and cryo-EM analysis of MHC-I molecules in different molecular contexts have gone some way to resolve.

By stabilising purified human PLCs with lipid nanodiscs before cryo-EM, Domnick et al visualised HLA A*03:01 molecules within an intact editing module (PLC-A*03:01 hereafter) with a maximum resolution of 3.7 angstroms [28]. This structure was complemented by two further structures, the first being that of MHC-I bound by a tapasin–ERp57 heterodimer [29]. The tapasin–ERp57–MHC-I structure was obtained by Muller et al, who employed an approach previously used to report the TAPBPR–H2-D^b^ structure [26], in which a combination of human and murine proteins enhanced the stability of the TAPBPR–MHC-I complex. To obtain tapasin–ERp57–MHC-I complexes, human tapasin–ERp57 heterodimers were mixed with UV-conditional peptide-loaded murine H2-D^b^ MHC-I molecules that had been refolded with human β_2_-microglobulin, before the peptide ligand was cleaved by UV exposure before crystallisation (tapasin–ERp57–H2-D^b^ hereafter). Second, Jiang et al provided a suite of structures, including MHC-I bound by tapasin [30]. The tapasin–MHC-I structure was obtained using an approach previously utilised to report the TAPBPR–H2-D^d^ structure [27], in which human tapasin was mixed with suboptimally peptide-loaded HLA-B*44:05 molecules (i.e. loaded with low-affinity peptides). The suboptimally loaded HLA-B*44:05 molecules were created using a C-terminally truncated peptide that was tethered by a disulphide bond to a cysteine introduced into the α1 helix at position 73, and which was further stabilised by an excess of F-pocket-binding dipeptide (tapasin-B*44:05-T73C hereafter).

**Figure 1:**
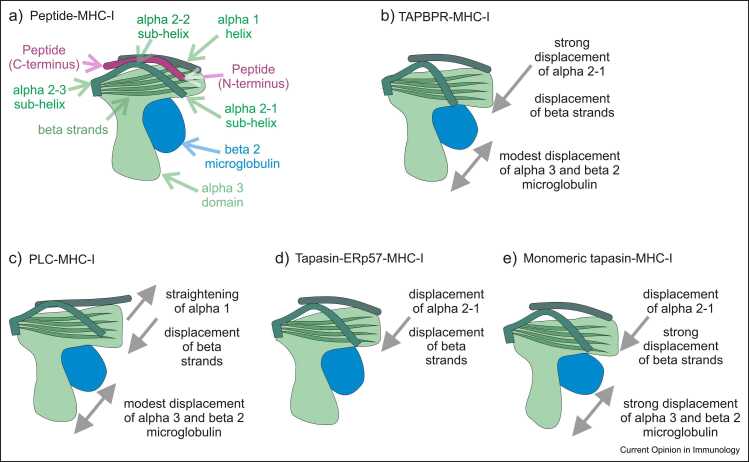
Representations of the differences in the crystal structures of tapasin-bound or TAPBPR-bound MHC-I molecules. Cartoon representations of the differences observed in the crystal structures of MHC-I molecules in different molecular contexts. **(a)** Annotation of key structural features of a peptide–MHC-I complex. **(b–e)** Changes observed in TAPBPR-bound MHC-I molecules **(b)**; PLC-bound MHC-I molecules **(c)**; tapasin–-ERp57 heterodimer-bound MHC-I molecules **(d)**; or monomeric tapasin-bound MHC-I molecules **(e)**. Arrows and text are used to describe the key differences that are discussed in the text.

These new structures complement the 2017 low-resolution PLC cryo-EM [5] and TAPBPR structures 26, 27. Comparison of these structures shows that while they share many similarities, they differ in key aspects (summarised in Figure 1). Before discussing how these structures may enhance our understanding of the MHC-I peptide-loading process, it is worth noting that these structures derive from crystals in which proteins are stabilised in low- energy states by crystal contacts. It is possible that an infrequently occupied, minor conformation may substantially misinform our understanding, particularly so if a protein is stabilised by crystal contacts in a non-physiologically relevant conformation. Additionally, it must be considered that a variety of biochemical approaches (discussed above) were employed to obtain peptide-receptive molecules that were stable enough to crystallise.

**Figure 2:**
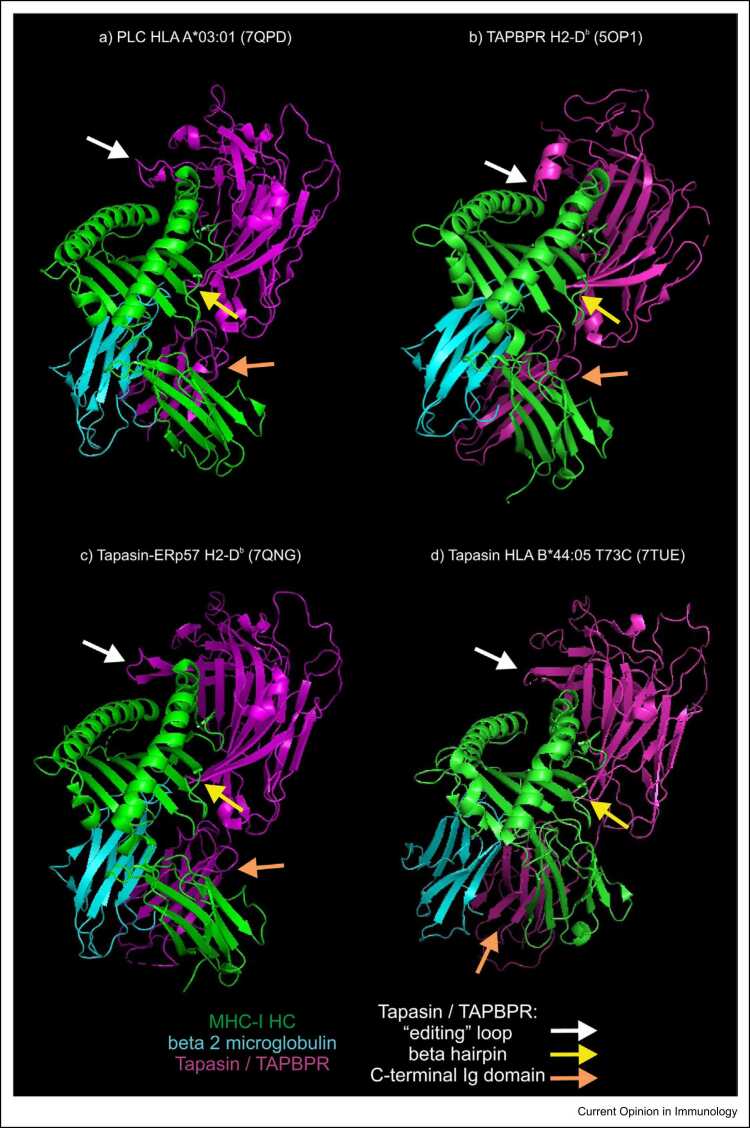
Structures of tapasin-bound or TAPBPR-bound MHC-I molecules. Structures depicting MHC-I molecules bound by either **(a)** tapasin as part of the PLC; **(b)** TAPBPR; **(c)** the tapasin–ERp57 heterodimer; **(d)** monomeric tapasin, with the structural features of tapasin or TAPBPR that are discussed in the text indicated by arrows. The MHC-I heavy chain is shown in green, β_2_-microglobulin is shown in cyan and tapasin or TAPBPR is shown in magenta. The structures of ERp57 and calreticulin are omitted from the PLC-A*03:01 and tapasin–ERp57–H2-D^b^ structures for clarity.

The structures show tapasin interacts with multiple surfaces of MHC-I, similar to the way TAPBPR binds MHC-I (Figure 2). Tapasin uses a concave three-tiered lumenal domain to bind MHC-I, cradling the MHC-I α2–1 sub-helix, with a β-hairpin supporting the peptide- binding domain, while the C-terminal immunoglobulin-like domain of tapasin nestles between the MHC-I α3 domain and β_2_-microglobulin. Comparison with the structure of non-MHC-I-bound tapasin–ERp57 heterodimers shows the β-hairpin and C-terminal immunoglobulin-like domain are repositioned relative to the N-terminal domain by binding MHC-I, suggesting tapasin can adopt multiple conformations that are likely to be important to facilitate peptide editing 5, 28••, 29••, 30••, 31.

In the PLC-A*03:01 and tapasin–ERp57–H2-D^b^ structures, residues 11–20 of tapasin form a loop, recently referred to by some investigators as the 'editing loop' 28••, 29•• that resides adjacent to the α-helices of the peptide-binding groove above the F pocket (Figure 2, white arrow). This loop was not visualised in the tapasin-B*44:05-T73C structure but was well resolved in an accompanying structure of an antibody-bound tapasin molecule (PDB 7TUF), revealing a similar conformation as in the PLC-A*03:01 and tapasin–ERp57–H2-D^b^ structures [30]. The structure adopted by these residues was not discernible without MHC-I, suggesting the loop was stabilised by binding MHC-I or anti-tapasin antibody.

The loop structures seen in the PLC-A*03:01, tapasin–ERp57–H2-D^b^ and antibody-bound tapasin structures are consistent with the findings of McShan and colleagues, who suggested this region of TAPBPR, which is five residues longer than in tapasin, adopted various non-helical conformations that hover above the peptide-binding groove and act as a peptide trap [32]. Others have previously proposed that this region of TAPBPR enters the peptide-binding groove (as a short alpha helix [26]) and competes against peptide for binding 33, 34.

The structures of ERp57 and calreticulin are not discussed in this review, apart from the interaction involving the N-linked glycan attached to N86 of MHC-I and the lectin-binding site of calreticulin. Mass spectrometry analysis showed that for nearly all of the MHC-I molecules within the PLC, two glucose molecules had been removed from the core (unprocessed) glycan initially attached to N86 [28]. Remarkably, cryo-EM analysis showed the carbohydrates of the A branch had a well-defined structure, stretching from N86 of MHC-I to the lectin-binding site of calreticulin [28]. In contrast, the structure of the mannoses in the B and C branches of the N-linked glycan could not be resolved, suggesting greater conformational flexibility [28]. Importantly, deglucosylation experiments showed that it was only once MHC-I was loaded with a high-affinity peptide that glucosidase II removed the last glucose from the N-linked glycan, suggesting that peptide loading and glucose trimming are allosterically coupled [28]. In these experiments, glucosidase II was able to deglucosylate MHC-I in the nanodisc-stabilised PLC, in which peptide loading of MHC-I did not lead to release from the PLC or the dissociation of calreticulin, suggesting that, despite its size, the PLC can accommodate significant molecular perturbations.

## The conformational plasticity of tapasin-bound or TAPBPR-bound major histocompatibility complex class-I

**Figure 3:**
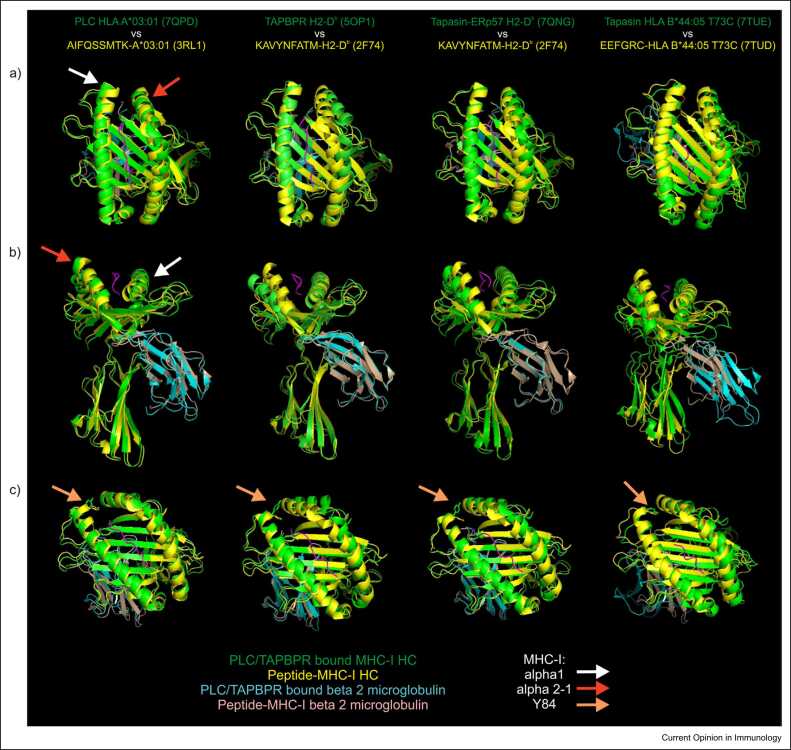
Comparisons of the peptide-binding domain of unchaperoned peptide-loaded MHC-I molecules with peptide-receptive MHC-I molecules bound by tapasin or TAPBPR. Models of tapasin-bound or TAPBPR-bound peptide-receptive MHC-I molecules were aligned with peptide-loaded, but unchaperoned MHC-I molecules. **(a)** Comparisons of the peptide-binding domain viewed from above. **(b)** Comparisons of the MHC-I molecules viewed from the front. **(c)** Comparisons of the side chains of Y84 of MHC-I. The MHC-I heavy chain is coloured green when bound by tapasin or TAPBPR, or yellow when unchaperoned. β_2_-microglobulin is coloured cyan when bound by tapasin or TAPBPR, and coloured wheat when unchaperoned. Arrows illustrate structural features discussed in the text. The structures of tapasin or TAPBPR, or other proteins present in the structures, are omitted for clarity.

The structures of tapasin-bound or TAPBPR-bound MHC-I molecules illustrate how MHC-I molecules adapt to different molecular partners. The most obvious difference concerned the α2–1 sub-helix, which TAPBPR dramatically rolled outwards and downwards, and which was also displaced along a similar trajectory by monomeric and ERp57-conjugated tapasin, although to a lesser degree (Figure 3, red arrow). In contrast, the α2–1 sub-helix was only modestly repositioned within the PLC. The substantial differences in the α2–1 sub-helices observed in these structures may reflect that this region is highly dynamic 24, 35•, 36, 37, and that different low-energy states were uniquely stabilised according to the molecular binding partner(s) or to the particular crystal form analysed.

The end of the α1 helix opposing the α2–1 sub-helix was straightened when MHC-I was incorporated into the PLC (Figure 3, white arrow), which may be a consequence of calreticulin interacting with the N-linked glycan attached to N86 of MHC-I. As a result of straightening of the α1 helix and the multiple interactions shared between tapasin and MHC-I, including residues of the 'editing' loop of tapasin, the distance between the α-helices flanking the empty F pocket increased. Notably, the hydrophobic side chain of leucine 18 (L18) of tapasin, a residue that can influence peptide binding to MHC-I 29••, 35•, points downwards between the α1 and α2–1 helices, forcing both outwards. Lan et al had proposed that lysine 16 (K16) of tapasin might stabilise MHC-I allotypes with acidic F pockets [35], however, the PLC-A*03:01 structure shows K16 is orientated away from peptide-binding groove and hydrogen bonds with asparagine 80 of the MHC-I α1 helix, rendering this proposal unlikely.

The orientation of several residues of MHC-I was changed in the different molecular environments, including tyrosine 84 (Y84), which usually hydrogen bonds with the MHC-bound peptide and stabilises the peptide–MHC-I complex 38, 39. In the PLC, Y84 of MHC-I was rotated outwards by L18 of tapasin (Figure 3c, orange arrow), allowing an interaction with glutamic acid 72 (E72) of tapasin, equivalent to the interaction with E102 of TAPBPR 26, 27, and Y84 was similarly rotated outwards in the tapasin–ERp57–H2-D^b^ structure. However, Y84 of MHC-I was not repositioned in the tapasin-B*44:05-T73C structure, with E72 of tapasin-binding arginine 145 of B*44:05-T73C instead [30]. The variable interactions involving Y84 are consistent with studies showing an interaction involving Y84 and tapasin, or Y84 and TAPBPR, is not obligatory for efficient MHC-I peptide loading 24, 32, 38. Indeed, while tapasin is found in all vertebrates, Y84 is only found in mammals and not in any other vertebrates 40, 41.

**Figure 4:**
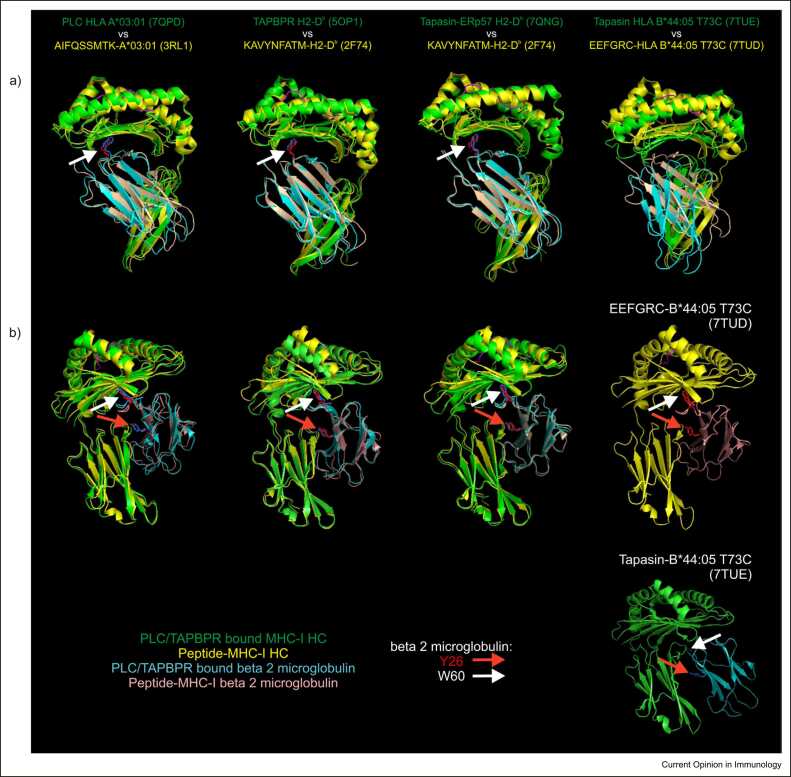
Comparisons of the α3 domain and of β_2_-microglobulin of unchaperoned peptide-loaded MHC-I molecules with peptide-receptive MHC-I molecules bound by tapasin or TAPBPR. Models of tapasin-bound or TAPBPR-bound peptide-receptive MHC-I molecules were aligned with peptide-loaded, but unchaperoned structures as in Figure 3. **(a)** Comparisons of the side chains of W60 of β_2_-microglobulin. **(b)** Comparisons of the side chains of Y26 and W60 of β_2_-microglobulin. The side chains of β_2_-microglobulin residues Y26 or W60 are coloured red for unchaperoned peptide-loaded molecules, or blue for tapasin-bound or TAPBPR-bound peptide-receptive MHC-I molecules. Owing to the substantial rearrangement of the α3 domain and of β_2_-microglobulin induced by monomeric tapasin, the location of W60 of β_2_-microglobulin is not shown in **(a)**, and the location of Y26 and W60 of β_2_-microglobulin is shown separately for the tapasin-bound and unchaperoned, peptide-bound molecules in **(b)**. The side chain of W60 of β_2_-microglobulin is not shown for the tapasin-B*44:05-T73C structure, because residues 56–63 could not be modelled accurately (dashed line) when bound by tapasin.

Another difference between the structures concerned the α3 domain and β_2_-microglobulin that were dramatically repositioned by monomeric tapasin (Figure 3, Figure 4) but were unaffected by the tapasin–ERp57 heterodimer and were modestly repositioned within the PLC (Figure 3, Figure 4). In the tapasin-B*44:05-T73C structure, the α3 domain, β_2_-microglobulin and the membrane-proximal immunoglobulin-like domain of tapasin are repositioned to form a trimer of immunoglobulin domains. Notably, this resulted in tyrosine 26 (Y26) and tryptophan 60 (W60) of β_2_-microglobulin being shifted away from the floor of the peptide-binding domain (W60, Figure 4b, white arrow) or further from the α3 domain (Y26, Figure 4b, red arrow) [42]. This reorientation is consistent with the inability to resolve β_2_-microglobulin residues 56–63 in structures when bound by tapasin, and by the pronounced line broadening of leucine 54 (L54) of β_2_-microglobulin in nuclear magnetic resonance spectra, suggesting L54 is sensitive to tapasin-bound and unbound states of MHC-I, and alternates between them when tapasin is present [30]. The repositioning of key residues such as Y26 and W60 in β_2_-microglobulin is likely to perturb the communication of dynamic motions between the peptide-binding groove and the α3 domain and β_2_-microglobulin, which modelling has proposed are key features of peptide editing 42••, 43.

Immunoprecipitation studies have indicated that all detectable tapasin is conjugated with ERp57 in vivo [44]. It is, therefore, reasonable to question the physiological relevance of the tapasin-B*44:05-T73C intermediate structure, particularly in light of the high tapasin independence of B*44:05 and the poor ability to co-immunoprecipitate B*44:05 with TAP or tapasin 2, 23, 43, 45; the major repositioning of the α3 domain and β_2_-microglobulin uniquely induced by monomeric tapasin; a substantial reduction in the surface area buried by the interaction with tapasin compared with the interaction with tapasin–ERp57 29••, 30••; and by the 'editing' loop of tapasin being unresolved in the tapasin-B*44:05-T73C structure, suggesting the absence of key stabilising tapasin–MHC-I interactions. Instead, this suggests that the multiple interactions between ERp57 and tapasin may restrain the dynamic properties of tapasin, preventing tapasin-bound MHC-I from sampling potentially non-peptide-receptive conformations, consistent with ERp57-conjugated tapasin functioning more effectively than monomeric tapasin [10].

## Conclusion

The structures provide tantalising views of how tapasin and TAPBPR exploit the plasticity of peptide-receptive MHC-I molecules to achieve peptide editing. Within the PLC, there is a subtle widening of the α-helices above the F pocket and mild displacement of the floor of the peptide-binding groove. In contrast, TAPBPR binding substantially deforms the α2–1 sub-helix and displaces the peptide-binding groove floor. When bound by either the PLC or TAPBPR, the α3 domain and β_2_-microglobulin are modestly repositioned. The wide-ranging nature of the conformational changes induced by binding tapasin or TAPBPR is entirely consistent with studies suggesting there is a coupling of dynamic motions both within the peptide-binding groove, including the A and F pocket 37, 39, and more widely throughout the MHC-I molecule 35•, 36, 42••, 43, 46.

**Figure 5:**
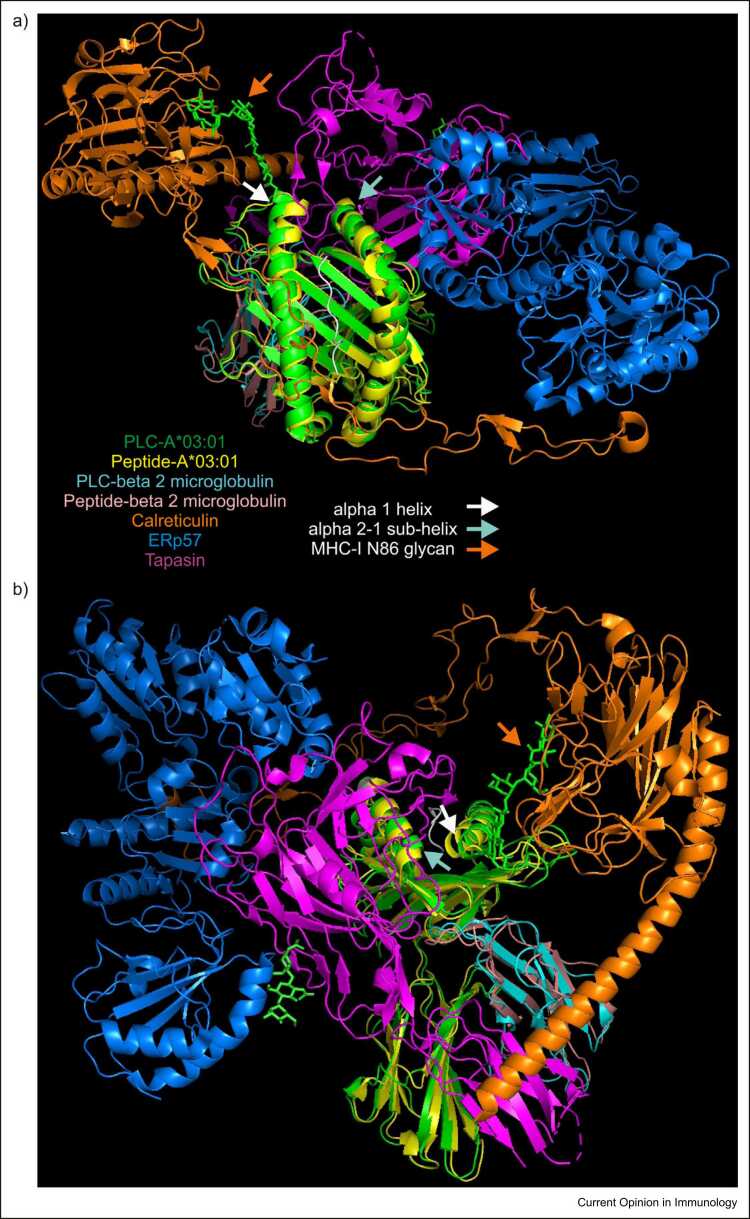
Comparison of an unchaperoned peptide-loaded MHC-I molecule with a peptide-receptive MHC-I molecule bound by tapasin within the PLC. A model of an unchaperoned peptide-A*03:01 complex (PDB 3RL1) was aligned with that of a PLC-bound peptide-receptive HLA A*03:01 molecule (PDB 7QPD), and viewed from above **(a)**, or from the side of the PLC **(b)**. The proteins are coloured as in the key, with the backbone of the peptide bound by the unchaperoned A*03:01 molecule shown in grey. When incorporated within a PLC, the distance between the - helices flanking the empty F pocket is widened because of calreticulin-induced straightening of the end of the α1 helix, and by residues of the 'editing' loop of tapasin pushing the α1 and α2–1 helices apart. The floor of the peptide-binding groove is supported by the β-hairpin of tapasin, while the α3 domain and β_2_-microglobulin are modestly repositioned by the membrane-proximal immunoglobulin-like domain of tapasin. Peptide binding decreases the highly excited dynamics of key residues within the MHC-I molecule, with dynamic information relayed throughout the MHC-I molecule via residues, including Y26 and W60 of β_2_-microglobulin. The affinity of the bound peptide is most efficiently sampled in the closed conformation, with release of peptides that do not form sufficient stabilising interactions with the MHC-I molecule allowing another attempt at peptide loading, or egress of peptide–MHC-I complexes through the secretory pathway. When loaded with a high-affinity peptide, the α1 and α2–1 sub-helices are pulled inwards, decreasing the distance above the now-occupied F pocket, releasing the MHC-I molecule from tapasin and releasing the glucose molecule of the N-linked glycan attached to N86 of the MHC-I molecule from the lectin-binding domain of calreticulin, permitting glucosidase II to remove the glucose molecule.

The PLC-A*03:01 and TAPBPR–H2-D^b^ structures likely illustrate alternative 'open' conformations of MHC-I molecules to which peptides readily bind, which have higher levels of free energy than 'closed' peptide-loaded molecules. It is possible that the differences between the PLC-A*03:01 and the tapasin–ERp57–H2-D^b^ structures may be a consequence of mixing human and murine proteins in the tapasin–ERp57–H2-D^b^ complex. However, the differences most likely indicate the influence that calreticulin has on the interactions within the PLC, including interactions with MHC-I, ERp57 and the membrane-proximal immunoglobulin-like domain of tapasin, which cumulatively restrains displacement of the α2–1 sub-helix (Figure 5). The greater deformation of MHC-I molecules induced by TAPBPR may reflect that TAPBPR has evolved to interact with MHC-I independently of the PLC and accordingly binds with higher affinity.

The structures show that tapasin and TAPBPR use multiple, broad and extensive interactions to deform MHC-I molecules. It is likely that tapasin and TAPBPR sample all MHC-I allotypes in a similar fashion, with the dynamic properties of the MHC-I allotypes dictating how much the peptide editors can enhance the sampling of structural states that are critical for peptide editing 24, 25, 28••, 29••, 32, 37, 43.

Peptide binding decreases the dynamics of key MHC-I residues 35•, 36, 37, 43, allowing the MHC-I molecule to transition to a stable, closed conformation in which the affinity of the peptide is most efficiently sampled. While the structural rearrangements of MHC-I within the PLC are subtle, they are sufficient to disrupt the interactions with tapasin, and to release the single glucose of the N-linked glycan attached to N86 of the MHC-I from the lectin-binding site of calreticulin sufficiently for glucosidase-II-mediated removal (Figure 5).

In conclusion, structural studies over the past three years have shed light on the importance of MHC-I protein dynamics for MHC-I peptide loading in the ER, showing how the MHC-I–β_2_-microglobulin heterodimer distorts as it binds different molecular partners. Some of the MHC-I structures incorporate peptides, or peptide fragments, which may give a glimpse of the encounter complex formed between MHC-I and an unedited peptide cargo (whose existence was inferred in 1998 — ref [7]). However, they do not provide a full molecular explanation for the phenomenon of peptide editing. Specifically, it is not known whether peptide repertoire sampling occurs by the intermediate state seen in the co-crystals or as we have suggested, by the native conformation following a peptide-induced conformational change triggered in the encounter complex. Nor do the recent structures explain MHC-I allele dependency on tapasin (and to a lesser extent TAPBPR). Specifically, it is not known whether tapasin- independent peptide editing follows a separate molecular mechanism, or whether tapasin- independent alleles can achieve the intermediate structure that is seen in the recent structures in the absence of tapasin (and TAPBPR). This is important because the majority of MHC-I alleles are able to present peptides to some extent via a tapasin-independent pathway (with MHC-I expression levels decreased by 10–90% in the absence of tapasin according to ref. [2]), and this is likely to impact on the composition of the peptide repertoire (i.e. the diversity and abundance of peptides) presented at the cell surface, and consequently the diversity of the CD8+ T-cell responses that they provoke.

## Declaration of Competing Interest

The authors declare that they have no known competing financial interests or personal relationships that could have appeared to influence the work reported in this paper.

## Data Availability

Data will be made available on request.
